# Human Zona Pellucida Glycoproteins: Binding Characteristics With Human Spermatozoa and Induction of Acrosome Reaction

**DOI:** 10.3389/fcell.2021.619868

**Published:** 2021-02-11

**Authors:** Satish Kumar Gupta

**Affiliations:** Reproductive Cell Biology Lab, National Institute of Immunology, New Delhi, India

**Keywords:** human zona pellucida glycoproteins, mutations in genes encoding human zona glycoproteins, zona proteins binding to sperm, ZP glycoproteins-mediated acrosome reaction, fertilization

## Abstract

Human zona pellucida (ZP) matrix is composed of four glycoproteins designated as ZP glycoprotein -1 (ZP1), -2 (ZP2), -3 (ZP3), and -4 (ZP4). Mutations in the genes encoding human ZP glycoproteins are one of the causative factors leading to abnormal ZP matrix and infertility in women. Relevance of the human ZP glycoproteins in ‘sperm–oocyte’ binding has been delineated by using either transgenic animal models expressing human zona proteins or purified native/recombinant human zona proteins. Studies based on the purified native/recombinant human zona proteins revealed that ZP1, ZP3, and ZP4 primarily bind to the capacitated acrosome-intact human spermatozoa whereas ZP2 binds to acrosome-reacted spermatozoa. On the contrary, human spermatozoa binds to the eggs obtained from transgenic mouse lines expressing human ZP2 but not to those expressing human ZP1, ZP3, and ZP4 suggesting that ZP2 has an important role in human ‘sperm–oocyte’ binding. Further studies using transgenic mouse lines showed that the N-terminus of human ZP2 mediate the taxon-specific human sperm–oocyte binding. Both glycans and protein-protein interactions have a role in human gamete interaction. Further studies have revealed that the purified native/recombinant human ZP1, ZP3, and ZP4 are competent to induce acrosome reaction. Human sperm binds to the mouse transgenic eggs expressing human ZP1-4 instead of mouse ZP1-3 proteins, penetrated the ZP matrix and accumulated in the perivitelline space, which were acrosome-reacted suggesting that human ZP2 in transgenic mouse model also induce acrosome reaction. In humans *N*-linked glycosylation of zona proteins have been shown to play an important role in induction of the acrosome reaction. Hence in humans, based on studies using transgenic mouse model as well as purified native/recombinant zona proteins, it is likely that more than one zona protein is involved in the ‘sperm–oocyte’ binding and induction of the acrosome reaction.

## Background

Zona pellucida (ZP), an extracellular glycoproteinaceous coat surrounding human oocyte is composed of four glycoproteins designated as zona pellucida glycoprotein -1 (ZP1), -2 (ZP2), -3 (ZP3), and -4 (ZP4). ZP glycoproteins play a critical role in ‘oogenesis,’ taxon-specific binding of the spermatozoa to the oocyte and induction of acrosome reaction (AR) in the spermatozoa bound to the ZP thereby facilitating accomplishment of fertilization. In essence, the ZP matrix serves as a ‘gate-keeper’ to regulate sperm binding (Hartmann et al., 1972). The human sperm binds to the human egg and do not bind with the eggs from other sub-hominoid primates such as baboon, rhesus monkey, and squirrel monkey as well as non-primate eutherian species (Bedford, 1977). The only exception to which human sperm binds is the oocytes of *Gorilla gorilla* and *Hylobates lar* (gibbon). Whereas, mouse sperm can bind to eggs from a taxonomically diverse group of mammals, including humans (Bedford, 1977). ZP glycoproteins also have a role in the prevention of polyspermy and ZP matrix protects the growing embryo till implantation.

In all eutherian mammals studied so far, ZP matrix has both ZP2 and ZP3. However, ZP1 and ZP4, which are encoded by paralogous genes probably formed by duplication of a common ancestral gene may or may not be present in all eutherian mammals (Goudet et al., 2008). For example, the ortholog of the human *ZP4* gene is present in the mouse genome as a pseudogene due to microdeletion of nucleotides leading to frame shift and appearance of a premature stop codon. Thus, functional ZP4 protein is not present in mouse ZP matrix (Lefievre et al., 2004; Goudet et al., 2008). Similarly, *ZP1* has been identified as a pseudogene in the dog and bovine genome (Goudet et al., 2008). High resolution Scanning Electron Microscopy (SEM) studies with human oocytes revealed that ZP appears as a delicate meshwork of thin interconnected filaments (Familiari et al., 1992, 2006). The filaments are 0.1–0.4 μm in length and 10–14 nm in thickness as observed by Transmission Electron Microscopy (Familiari et al., 1992). In mouse ZP, the filaments are composed of ZP2 and ZP3 heterodimers, which are cross-linked by ZP1 homodimers (Greve and Wassarman, 1985; Green, 1997). The precise arrangement of ZP filaments in the human ZP matrix has not been deciphered. However, supramolecular structure based on the structural information of ZP glycoproteins has been proposed for human ZP (Bokhove and Jovine, 2018). It comprises of filaments with a structural repeat of ∼14 nm formed by alteration of ZP3 and either ZP2 or ZP4. ZP1 will be occasionally incorporated instead of ZP2/ZP4 and stabilizes the ZP by intermolecular cross-links between filaments (Bokhove and Jovine, 2018). Recently, it has been shown that ZP filaments are indeed cross-linked by ZP1 homodimers leading to the formation of a stable matrix (Nishimura et al., 2019). Meshwork of filaments leads to formation of the pores that appear larger at the outer surface of the zona than the inner surface. Ultrastructural cytochemical findings further suggested that the porous region of the human ZP is limited to ∼25% of the external region of the human ZP, while the compact region constitutes ∼75% of the total ZP. The amorphous spongy outer surface of zona with larger pores may facilitate sperm penetrability as human ZP with a more compact and smoother outer surface has been shown to be less penetrable (Familiari et al., 1988, 1992). On the contrary, no correlation was observed between the ZP morphology and success of *in vitro* fertilization (Magerkurth et al., 1999).

In this ‘review,’ characteristics of the respective human ZP glycoproteins will be briefly described. The role of mutations in the genes encoding human ZP glycoproteins leading to morphologically abnormal oocytes and infertility will be discussed. The relevance of human ZP glycoproteins for binding with human spermatozoa and their ability to induce AR as observed by using either transgenic animal models or purified native/recombinant proteins will be described.

## Expression Profile of the ZP Glycoproteins in Human Oocytes

Expression of human ZP3 has been observed in the oocytes of fetal ovary (Törmälä et al., 2008). By immunohistochemistry, expression of human ZP1, ZP2, and ZP3 has been observed in oocytes as well as granulosa cells of the primordial follicles (Gook et al., 2008). Expression of ZP4 has been described in the zona of mature oocytes (Lefievre et al., 2004). Contrary to these observations, employing highly specific mouse monoclonal antibodies raised against recombinant human ZP2, ZP3, and ZP4 and synthetic peptide (219–258 aa residues) corresponding to human ZP1, expression of human ZP1, ZP2, and ZP3 is observed in oocytes of growing and antral follicles (Bukovsky et al., 2008; Ganguly et al., 2010b). Expression of ZP4 is observed in oocytes of primordial, growing and antral follicles (Bukovsky et al., 2008). Expression of ZP1 and ZP2 though not observed in oocytes of primordial follicles but it is observed in oocytes of primordial follicles undergoing atresia (Bukovsky et al., 2008; Ganguly et al., 2010b). Employing human cumulus oocyte complexes, it has been shown that the expression of *ZP1*, *ZP2*, and *ZP4* at the transcript level is higher in immature (Metaphase 1, M1/Germinal Vesicle, GV) oocytes as compared to mature (Metaphase II, MII) oocytes. This coincides with the smaller inner layer-ZP area and thickness in the mature as compared to immature oocytes (Canosa et al., 2017). These observations suggest that the nascent ZP glycoproteins are incorporated into the inner surface of the ZP matrix. Hence, thickening of the ZP matrix takes place from inside to the outside.

## Genomic Organization of Human ZP Glycoproteins and Functional Significance of the Common Structural Motifs

The human genome contains four *ZP* genes: *ZP1*, *ZP2*, *ZP3*, and *ZP4*, which are located on chromosomes 10, 16, 7, and 1, respectively (Hughes and Barrat, 1999).

### Human ZP1

The human *ZP1* gene has 12 exons and encodes a polypeptide of 638 amino acid (aa) (Lefievre et al., 2004). Expression of *ZP1* mRNA is low in the fetal as well as adult ovaries (Törmälä et al., 2008). Human ZP1 has a 25 aa long signal peptide, 41 aa long ‘trefoil domain,’ signature ‘ZP domain’ from 279 to 548 aa and consensus furin cleavage site (CFCS), RQRR, from 552 to 555 aa. ‘ZP domain’ of human ZP1 consists of two subdomains, ZP-N and ZP-C. From human ZP1-N subdomain, two ‘aggregation-prone’ peptides have been predicted that may be crucial for ZP protein polymerization as these peptides self-assemble into amyloid-like fibrils (Louros et al., 2013). In humans, filaments are cross-linked by ZP1-N subdomain of ZP1 leading to the formation of stable ZP matrix, which can be modulated by ZP1 fucosylation and zinc sparks (Nishimura et al., 2019). At aa level, human ZP1 has 47% identity with human ZP4 suggesting that these two proteins may have evolved from a common ancestral gene either by gene duplication or exon swapping.

### Human ZP2

The human *ZP2* gene has 19 exons and encodes a 745 aa long polypeptide. ZP2 has 38 aa long signal peptide, ‘ZP domain’ from 372-637 aa and CFCS, RHRR from 639 to 642 aa.

### Human ZP3

The human *ZP3* gene has 8 exons and encodes a 424 aa long polypeptide. It has a 22 aa long signal peptide, ‘ZP domain’ corresponding to aa residues 45–303 and CFCS, RNRR from 349 to 352 aa. In humans, second polymorphic locus for human *ZP3* encoding a truncated protein corresponding to 372 aa residues in addition to full-length ZP3 has also been reported (van Duin et al., 1992). In addition, a bipartite RNA transcript encoded by *POM-ZP3* gene that is derived from a gene homologous to rat *POM121* (encode a nuclear pore membrane protein) and 4 C-terminal exons of human *ZP3* has also been reported in humans (Kipersztok et al., 1995).

### Human ZP4

The human *ZP4* gene has 13 exons and encodes a 540 aa long polypeptide with 18 aa long signal peptide, ‘trefoil-domain’ corresponding to 141–183 aa residues, ‘ZP domain’ corresponding to 188–460 aa residues and CFCS, SRRR, from 463 to 465 aa residues. Self-assembly of peptides corresponding to a common interface of human ZP2, ZP3, and ZP4 into fibrils with distinct amyloid-like features have been reported. It suggests that ‘ZP domain’ plays an important role in polymerization and self-assembly of ZP glycolproteins (Louros et al., 2016).

The deduced amino acid (aa) sequence of the four human ZP glycoproteins revealed some common structural elements, which plays an important role in their secretion, incorporation in the ZP matrix, structure and function. All four human ZP glycproteins have N-terminal hydrophobic signal peptide that targets them to the secretory pathway through co-translational import into the endoplasmic reticulum and which ultimately gets cleaved from the mature proteins by signal peptidase present in the oocytes. Human ZP1 and ZP4 have ‘trefoil domain,’ which is absent in ZP2 and ZP3. ‘Trefoil domain’ has a characteristic pattern of 6 conserved cysteine in a trefoil-like arrangement and is found in a family of small peptides called the Trefoil family (Thim, 1989). The structural and/or functional significance of its presence in human ZP1 and ZP4 is not clear. All four human ZP glycoproteins share a motif designated as the ‘ZP domain,’ which consists of approximately 260 aa including 8 conserved cysteine residues and is predicted to have high β-strand content with additional conservation of hydrophobicity, polarity, and turn-forming tendency (Bork and Sander, 1992; Jovine et al., 2005). It has a bipartite structure with ZP-N and ZP-C subdomains separated by a protease-sensitive region. ‘ZP domain’ plays an important role in the polymerization of human zona proteins into filaments (Jovine et al., 2002). Immediately after ‘ZP domain’ all four human ZP glycoproteins have CFCS. Proteolytic cleavage at CFCS by proprotein convertase enzyme is critical for the secretion and assembly of human ZP3 in ZP matrix (Kiefer and Saling, 2002). The importance of CFCS in the secretion and assembly of other human zona proteins is still awaited. It is also not clear if the cleavage takes place either in the Golgi or at the egg plasma membrane. Downstream of CFCS, hydrophobic transmembrane-like domain (TMD) and short cytoplasmic tail is present in all the four human ZP glycoproteins. The functional significance of TMD and cytoplasmic tail for human ZP glycoproteins is not known. However, cytoplasmic tails of mouse ZP2 and ZP3 prevent their premature intracellular interaction and thus plays an important role in the incorporation of ZP2 and ZP3 into the ZP matrix (Jimenez-Movilla and Dean, 2011).

## Biochemical Characteristics of Human ZP Glycoproteins

Iodination and subsequent two-dimensional SDS-PAGE analysis of the heat solubilized isolated zona pellucida (SIZP) from human eggs showed three acidic proteins with molecular weights ranging from 64 to 78 kDa (ZP2), 57 to 73 kDa (ZP3), and 90 to 110 kDa (ZP4; previously classified as ZP1) (Shabanowitz and O’Rand, 1988). Using antibodies generated against synthetic peptides, ZP2 has been characterized as 90–110 kDa and ZP3 as 53–60 kDa glycoproteins (Bauskin et al., 1999). Using highly specific mouse monoclonal antibodies raised against recombinant human ZP2, ZP3, and ZP4, these proteins have been purified from the human eggs by immunoaffinity column chromatography. The purified native human ZP2, ZP3, and ZP4 (also contaminated with ZP1) revealed ∼120, ∼58, and ∼65 kDa bands respectively in denaturing SDS-PAGE (Chiu et al., 2008b). Based on deduced aa sequence, the calculated molecular weight of human ZP1, ZP2, ZP3, and ZP4 is 57, 82, 47, and 59 kDa respectively. The higher molecular weight of the purified native human ZP2, ZP3, and ZP4 from the human eggs as compared to the calculated molecular weight of the respective protein may be due to glycosylation. Analyses of human ZP by 2-D SDS-PAGE and Western blots revealed that ZP2, ZP3, and ZP4 showed multiple isoforms, which may be due to varying extend of glycosylation (Bercegeay et al., 1995; Gupta et al., 1998). Using lectins, high concentration of D-mannose residues have been reported in the human ZP (Maymon et al., 1994). Further characterization using lectins and antibodies revealed the presence of sialyl-Lewis^*a*^, sialyl-Lewis^*x*^, Neu5Acα2-3Galβ1,4Glc-NAc, Galβ1,3GalNAc-Ser/Thr, Neu5Acα2,6Gal/GalNAc, fucosylated oligosaccharides, *N*-acetylgalactosamine residues, galactose residues, and *N*-acetylglucosamine residues in the ZP matrix of human oocytes (Jiménez-Movilla et al., 2004). Further analyses of purified human ZP2, ZP3, and ZP4 subsequent to either *N*-glycosidase-F treatment (removal of *N*-linked oligosaccharides) or alkaline reduction (removal of *O*-linked oligosaccharides) suggested that *N*-linked glycosylation occupies ∼37%, ∼27%, and ∼18% of the molecular mass of ZP2, ZP3, and ZP4, respectively (Chiu et al., 2008b). Human ZP2 has ∼8% and ZP3 has ∼9% *O*-linked glycosylation. Alkaline reduction of purified human ZP4 did not lead to any significant reduction in its electrophoretic mobility in denaturing SDS-PAGE suggesting that it either has no *O*-linked glycosylation or minimally *O*-linked glycosylated (Chiu et al., 2008b). These observations clearly show that human ZP glycoproteins have more *N*-linked as compared to *O*-linked glycosylation. Sialyl-Lewis^*x*^sequence [NeuACα2-3Galβ1-4(Fucα1-3)GlcNAc] is the most abundant terminal sequence on the *N*- and *O*-glycans as revealed by the mass spectrometric analysis of the human ZP (Pang et al., 2011).

## Mutations in Genes Encoding Human Zona Proteins: Probable Causative Factor for Infertility in Women

Analysis of *ZP1*, *ZP2*, *ZP3*, and *ZP4* genes in women whose eggs fail to fertilize using *in vitro* fertilization (IVF) as compared to those with successful fertilization following IVF as well as women with proven fertility showed 1.5-fold increase in sequence variation in *ZP1* and *ZP3* genes (Männikkö et al., 2005). An additional study in infertile women revealed sequence variations in genes encoding *ZP2* and *ZP3* (Pökkylä et al., 2011). Analysis of the nucleotide sequence of *ZP1* gene from six members of the family (five sisters and one brother; four sisters diagnosed with primary infertility; two out of four sisters had eggs which were not surrounded with ZP matrix, other two sisters had no eggs) revealed homozygous frameshift mutations inherited in an autosomal recessive mode, which led to premature stop codon and resulted in a truncated ZP1 (404 aa residues), which was postulated to sequester ZP3 in the cytoplasm and prevented the formation of ZP matrix (Huang et al., 2014). Two mutations in the gene encoding *ZP1* [one missense variant c.247T > C (p.W83R) and one nonsense variant c.1413G > A (p.W471X)] have also been reported from infertile women who had oocytes with morphological defects (Yang et al., 2017). The nonsense variant c.1413G > A is located in the ‘ZP domain’ and leads to premature stop codon resulting in truncation of the ZP1 protein from 638 aa to 471 aa (Yang et al., 2017). In the same study, another 33-year-old woman showed *ZP2* variant c.1696T > C in exon 16 leading to change of cysteine with arginine at position 566 (p.C566R). In another 28-year-old woman, variant c.1599G > T in exon 15 of *ZP2* that led to the replacement of arginine to serine (p.R533S) has also been reported (Yang et al., 2017). Two novel heterozygous mutations in *ZP2* (c.2092C > T; arginine at 698^th^ position replaced by a stop codon) and *ZP3* (c.1045_1046 insT; arginine at 349^th^ position replaced by L amino acid followed by a stop codon) from a woman and her family members with abnormal ZP have also been reported (Liu et al., 2017). Further studies in mouse model using CRISPR/Cas9 gene editing technology revealed that oocytes obtained from mice with either of the heterozygous mutations showed thinner ZP as compared to the oocytes obtained from wild mice. Interestingly, oocytes from female mice with both mutations showed even thinner ZP as compared to the ZP of oocytes with single mutation or absence of ZP suggesting that these mutations have dosage effect with respect to the formation of ZP (Liu et al., 2017). Both the mutant proteins failed to anchor on the oocyte membrane. A paternally transmitted heterozygous missense mutation of c.400 G > A (p.A134T) in *ZP3* in women with empty follicle syndrome has also been reported. Immunofluorescence and histological analysis revealed degenerated oocytes and some of which were devoid of ZP matrix (Chen et al., 2017). In another recent study, seven patients belonging to six independent families with abnormal oocytes or those suffered from empty follicle syndrome revealed three homozygous mutations in *ZP1* [c.1708G > A (p.V570M), c.1228C > T (p.R410W), c.507del (p.H170Iefs^∗^52)], two mutations in a compound heterozygous state in *ZP1* [c.1430 + 1G > T (p.C478X), c.1775-8T > C (p.D592Gfs^∗^29)], a homozygous mutation in *ZP2* [c.1115G > C (p.C372S)], and a heterozygous mutation in *ZP3* [c.763C > G (p.R255G)]. Interestingly, expression studies of human ZP1, ZP2, and ZP3 with these mutations in CHO cells showed defects in their expression, secretion, and interaction suggesting that these mutations are responsible for the abnormal oocyte phenotype observed in these patients (Zhou et al., 2019). Two homozygous mutations in *ZP2* [c.1695-2A > G and c.1691_1694dup (p.C566Wfs^∗^5)] have also been reported in women with a thin ZP and defective sperm binding to the oocyte from two unrelated consanguineous families (Dai et al., 2019). Expression studies in CHO cells led to truncated ZP2 protein. Whole-exome sequencing of *ZP1* in two infertile sisters from a family with empty follicle syndrome revealed compound heterozygous mutations. Co-immunoprecipitation studies and homology modeling analysis showed that both mutated ZP1 disrupt the formation of oocyte ZP by interrupting the interaction among ZP1, ZP2, and ZP3 (Sun et al., 2019). These studies suggest that deleterious mutations in the genes encoding human ZP glycoproteins are one of the causative factors for female infertility due to defective ZP and leading to failure of fertilization.

## Role of Human ZP Glycoproteins in Binding to the Spermatozoa and Induction of Acrosome Reaction

Human ZP matrix is composed of four glycoproteins whereas mouse ZP matrix is composed of three glycoproteins. It is pertinent to know, if the role of respective human ZP glycoproteins in sperm–oocyte binding and induction of AR is same as deciphered in mouse model or additional proteins are involved during these steps to accomplish fertilization. In mouse model, the function of individual ZP glycoproteins during fertilization has been elucidated with ‘loss-of-function’ by targeted mutagenesis of individual ZP genes in embryonic stem cells to generate null mutant mice defective for one of the zona proteins. Further, transgenic mouse lines expressing human ZP proteins have been developed to study the binding of human sperm to transgenic mouse eggs that is ‘gain-of-function’ (Avella et al., 2013). In addition to transgenic animal models, purified native/recombinant human ZP proteins have also been used to delineate their biological functions during sperm-egg binding and induction of AR (Gupta et al., 2012; Gupta, 2018). The salient findings with respect to the role of various ZP glycoproteins during sperm-egg binding and induction of AR will be described below using both these approaches.

### Human ZP1

#### Studies Using Transgenic Mouse Model Revealed No Role of ZP1 in Sperm-Egg Binding

To delineate the role of ZP1 during fertilization, *ZP1*-null mouse lines have been developed by targeted mutagenesis of *ZP1* in embryonic stem cells (Rankin et al., 1999). In *ZP1* null mice, the ZP is composed of only mouse ZP2 and ZP3 and the matrix is more loosely organized than zonae around normal oocytes. These mice have perturbed folliculogenesis and after mating with males, fewer two-cell embryos are recovered from *ZP1* null mice. Hence, mouse ZP1 is not essential for sperm binding or fertilization but it is required for structural integrity of the ZP matrix to minimize precocious hatching and reduced fecundity (Rankin et al., 1999). Subsequently, to delineate the role of human ZP1 in sperm-egg binding, transgenic mouse expressing human ZP1 was crossed with ZP1-null background mouse to produce transgenic mouse lines with zonae expressing human ZP1, mouse ZP2, and mouse ZP3 (Baibakov et al., 2012). The eggs obtained from these transgenic mice failed to bind human sperm suggesting that ZP1 may not be involved in human sperm-egg binding (Baibakov et al., 2012).

#### Recombinant Human ZP1 Binds to the Capacitated Human Spermatozoa and Induces AR

**FIGURE 1:**
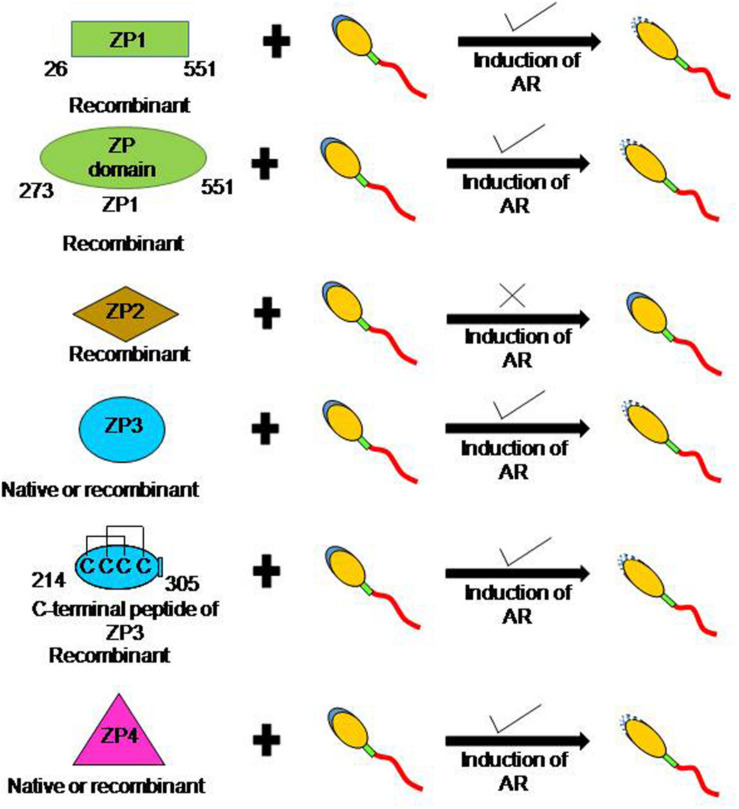
Schematic diagram to depict the ability of human ZP glycoproteins to induce acrosome reaction. In humans, more than one zona protein is competent to induce AR. Incubation of capacitated human sperm with baculovirus-expressed recombinant ZP1, ‘ZP domain’ of ZP1 (273–551 aa residues), ZP3, C-terminal fragment of ZP3 (214–305 aa residues), and ZP4 led to a dose dependent increase in AR. Further, several studies have also shown that recombinant human ZP3 expressed in mammalian cells also induce AR. In addition, induction of AR has also been observed in presence of native ZP3 and ZP4 purified from human eggs. However, baculovirus-expressed recombinant ZP2 failed to induce AR.

As of today, there is no report of purification of the human ZP1 from human eggs. However, human ZP1 (26–551 aa residues) without signal peptide sequence and till the CFCS has been expressed in *E. coli* as well as in insect cells (Ganguly et al., 2010b). Both baculovirus- and *E. coli*-expressed recombinant human ZP1 binds to the capacitated acrosome-intact human sperm (Table 1). In calcium ionophore-induced acrosome-reacted human sperm, ZP1 binding to the acrosomal cap is lost and its binding is restricted to the equatorial region only. In an additional study, the importance of ‘ZP domain’ of ZP1 for binding to the capacitated spermatozoa has also been shown (Ganguly et al., 2010a; Table 1). Baculovirus-expressed recombinant human ZP1 (273–551 aa residues) encompassing ‘ZP domain’ of ZP1, showed binding profile with capacitated acrosome-intact and acrosome-reacted human sperm similar to that observed with recombinant human ZP1 (26–551 aa residues). Interestingly, incubation of the capacitated human sperm with baculovirus-expressed recombinant human ZP1 (26–551 aa residues) as well as recombinant protein corresponding to ‘ZP domain’ of ZP1 (273–551 aa residues) led to a dose dependent significant increase in the AR (Ganguly et al., 2010a,b; Figure 1). Approximately 30% sperm undergo AR in presence of recombinant human ZP1 (26–551 aa residues; 10 μg/ml) and ∼40% in presence of ZP domain of ZP1 (5 μg/ml) as compared to ∼8% either spontaneous AR or in presence of Fetuin (Ganguly et al., 2010a,b). However, *E. coli*-expressed recombinant human ZP1 (26–551 aa residues), though binds to the capacitated spermatozoa but failed to induce any significant increase in the AR. These observations suggest that glycosylation of human ZP1 is important for induction of the AR. The studies using recombinant human ZP1 suggests that it has a role in human sperm-egg binding, which are contrary to the observations from transgenic mouse model (Table 2).

**TABLE 1:** Binding profile of human zona pellucida glycoproteins to the capacitated acrosome-intact and acrosome-reacted human sperm.

Human Zona Protein	Binding profile of ZP protein with		References
	
	Capacitated acrosome-intact sperm	Capacitated acrosome-reacted sperm	
*E. coli*-/baculovirus-expressed recombinant ZP1 (26–551 aa)	Acrosomal cap, Equatorial region	Equatorial region	Ganguly et al., 2010b
Baculovirus-expressed recombinant ‘ZP domain’ of ZP1 (273–551 aa)	Acrosomal cap, Equatorial region	Equatorial region	Ganguly et al., 2010a
*E. coli*-expressed recombinant ZP2 (39–242 aa)	No specific binding	Head, Midpiece	Tsubamoto et al., 1999
*E. coli* (38–645 aa)-/baculovirus (1–745 aa)-expressed recombinant ZP2	No specific binding	Equatorial region	Chakravarty et al., 2008
Native ZP2 purified from human eggs	Equatorial and post-acrosomal regions, Midpiece	Acrosomal, equatorial and post-acrosomal regions, Midpiece	Chiu et al., 2008b
*E. coli* (23–348 aa)-baculovirus (1–424 aa)-expressed recombinant ZP3	Acrosomal cap, Equatorial region	Equatorial region	Chakravarty et al., 2008
Native ZP3 purified from human eggs	Acrosomal and Equatorial regions, Midpiece	Midpiece	Chiu et al., 2008b
Baculovirus-expressed recombinant ZP3 fragments (23–175, 1–370, 214–348, 214–305 aa)	Anterior head, Equatorial region	Equatorial region	Bansal et al., 2009
*E. coli* (22–463 aa)- and baculovirus (1–540 aa)-expressed recombinant ZP4	Acrosomal cap, Equatorial region	Equatorial region	Chakravarty et al., 2008
Native ZP4 purified from human eggs	Head region	No binding to head region	Chiu et al., 2008b

**TABLE 2:** Salient findings on the role of human ZP glycoproteins in sperm-egg binding and induction of AR using either transgenic animal models or native/recombinant proteins.

Human zona pellucida protein	Functional aspect of human ZP glycoproteins by using	
	
	Transgenic animals	Purified proteins
ZP1	Eggs from transgenic mouse lines with zonae expressing human ZP1, mouse ZP2, and mouse ZP3 failed to bind human sperm suggesting no role for human ZP1 in sperm-egg binding	Baculovirus-expressed recombinant human ZP1 binds to the capacitated human spermatozoa and induce dose-dependent AR
ZP2	Eggs from transgenic mouse lines with zonae expressing human ZP2, mouse ZP1, and mouse ZP3 as well as those expressing human ZP1-4 in place of mouse ZP1-3 bind human sperm and sperm accumulated in perivitelline space have undergone AR. The spermatozoa binding site resides in the N-terminal fragment of ZP2. ZP2 plays an important role in sperm-egg binding.	Human sperm bind to beads coated with recombinant human ZP2 peptide (39–154 aa residues). Other studies using native/recombinant protein revealed ZP2 binding to acrosome-reacted human spermatozoa and its inability to induce AR in capacitated human sperm.
ZP3	Eggs from transgenic mouse lines with zonae expressing human ZP3, mouse ZP1, and mouse ZP2 do not bind human sperm suggesting that ZP3 has no role in sperm-egg binding.	Native/recombinant ZP3 binds to the capacitated human spermatozoa and numerous studies showed that it induces AR in capacitated human spermatozoa.
ZP4	Eggs from transgenic mouse lines with zonae expressing human ZP4, mouse ZP1-3 failed to bind human sperm suggesting no role for human ZP4 in taxon-specific sperm-egg binding	Native/recombinant ZP4 binds to the capacitated human spermatozoa and induce AR.

### Human ZP2

#### Transgenic Mice Studies Revealed the Role of ZP2 in Sperm-Egg Binding

To delineate the role of ZP2 during fertilization, *ZP2* knock-out transgenic mouse lines have been developed. In *ZP2* null mice, a thin ZP matrix in early follicles with mouse ZP1 and ZP3 synthesis has been observed that could not be sustained in pre-ovulatory follicles (Rankin et al., 2001). The abnormal ZP matrix does not affect initial folliculogenesis, but there is a significant reduction in the number of antral stage follicles. No 2-cell embryos are recovered after mating *ZP2* null females with normal male mice, suggesting that mouse ZP2 has a role during fertilization and early embryo development (Rankin et al., 2001). Further, transgenic mouse lines expressing mouse ZP1, mouse ZP3 and human ZP4 but not mouse ZP2 have also been developed with normal appearing ZP matrix (Avella et al., 2014). The eggs obtained from these transgenic mice lacking expression of mouse ZP2 failed to bind mouse sperm suggesting that mouse ZP2 is required for sperm-egg binding and fertility (Avella et al., 2014). In mice, post-fertilization ZP2 undergoes proteolytic cleavage by oocyte-specific ovastacin – a metalloendoprotease leading to changes in the supramolecular structure of the ZP matrix and thereby prevent polyspermy (Burkart et al., 2012). If the ovastacin cleavage site (^166^LA DE^169^) was mutated in transgenic mice or ovastacin was genetically ablated, ZP2 does not undergo cleavage and sperm continued to bind early embryo (Gahlay et al., 2010; Burkart et al., 2012). Additional experiments are needed to show whether ZP2 also has a role in avoidance of polyspermy during fertilization in humans.

**FIGURE 2:**
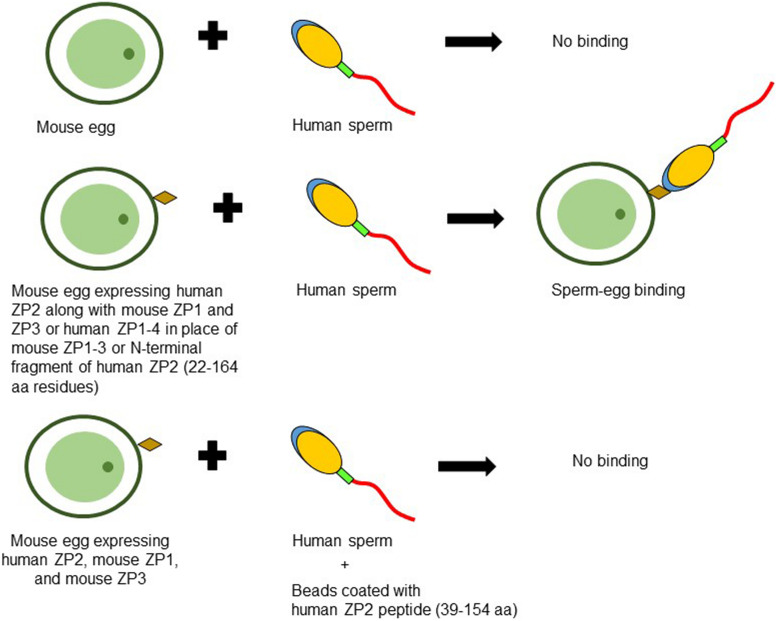
Schematic diagram to illustrate binding of human sperm to transgenic mouse oocyte expressing human ZP2. No binding of human capacitated sperm is observed when incubated with mouse eggs expressing mouse ZP1, ZP2, and ZP3. However, zonae from transgenic mouse lines expressing either human ZP2, mouse ZP1, and mouse ZP3 or human ZP1-4 in place of mouse ZP1-3 or those expressing human ZP2 N-terminal fragment corresponding to 22–164 aa residues instead of mouse ZP2, showed binding with human sperm suggesting its important role in human sperm-egg binding. Agarose beads coated with human ZP2 peptide (39–154 aa residues) inhibited the binding of human sperm to transgenic mouse oocytes expressing human ZP2 (Baibakov et al., 2012; Avella et al., 2014, 2016).

Subsequently, transgenic mouse lines wherein mouse ZP2 and/or ZP3 were replaced with human homologs have been developed (Rankin et al., 2003). The eggs obtained from transgenic mice expressing human ZP2, mouse ZP1, and mouse ZP3 failed to bind human sperm (Rankin et al., 2003). However, eggs from these transgenic mice showed *in vitro* binding of mouse sperm leading to successful fertilization and formation of two-cell embryo. After fertilization, human ZP2 failed to be cleaved by ovastacin and mouse sperm showed continued binding with early rescued embryos (Rankin et al., 2003). Further, four transgenic mouse lines expressing human ZP1, ZP2, ZP3, and ZP4 were crossed to establish transgenic mouse line expressing human ZP1-4 in place of mouse ZP1-3 (Baibakov et al., 2012). *In vitro* sperm-egg binding assay revealed that human sperm bind to the transgenic zonae expressing human ZP2, mouse ZP1, and mouse ZP3 as well as transgenic zonae expressing human ZP1-4 proteins in place of mouse ZP1-3 (Baibakov et al., 2012; Avella et al., 2014; Figure 2). The failure to observe human sperm binding to the transgenic zonae expressing human ZP2, mouse ZP1 and mouse ZP3 in the previous study (Rankin et al., 2003) was due to the insufficient time allowed for capacitation of human sperm (Baibakov et al., 2012; Avella et al., 2014). In case of transgenic eggs expressing human ZP1-4, human sperm not only bind to the eggs but also penetrated ZP matrix and accumulated in the perivitelline space (Avella et al., 2014). The sperm accumulated in perivitelline space are acrosome-reacted. Using DNA recombinant technologies and pronuclear injection, transgenic mouse lines expressing human exons encoding human ZP2 (22–164 aa residues) and mouse exons encoding mouse ZP2 (18–156 aa residues) either as hu/moZP2 or mo/huZP2 configuration have been developed. In addition, zonae from the above transgenic mouse lines also expressed mouse ZP1 and ZP3. The ZP of the chimeric hu/moZP2 eggs reacted with human ZP2 N-terminus and mouse ZP2 C-terminus but not mouse ZP2 N-terminus recognizing monoclonal antibodies. The ZP surrounding the chimeric mo/huZP2 eggs reacted with antibodies to the N-terminus but not the C-terminus of mouse ZP2. Human sperm bind to the chimeric zonae expressing hu/moZP2 but not to those expressing mo/huZP2 chimeric protein thereby suggesting that the N-terminus of ZP2 mediate the taxon-specificity of human sperm binding to the ZP (Avella et al., 2014).

#### Human ZP2 Protein/Fragment Binds to Either Capacitated or Acrosome-Reacted Human Sperm

Contradictory observations about the ability of native and/or recombinant human ZP2 for binding to the capacitated human spermatozoa have been reported. It has been demonstrated that agarose beads coated with recombinant human ZP2 peptides corresponding to either 39–154 or 39–267 aa residues bind specifically with human sperm (Baibakov et al., 2012; Avella et al., 2016). Interestingly, human ZP2 peptide (39–154 aa residues) coated beads prevented the binding and penetration of the human sperm to the transgenic eggs expressing human ZP2 in place of mouse ZP2 (Avella et al., 2016; Figure 2). Using immobilized baculovirus-expressed recombinant human ZP2 in a solid-phase immunoassay, binding of capacitated human sperm has been reported with ZP2, though it was lower as compared to immobilized ZP4 and ZP3 (Chirinos et al., 2011).

On the other hand, binding studies with *E. coli*-expressed N-terminal fragment of recombinant human ZP2 (39–242 aa residues, excluding signal peptide) revealed that it does not bind to the capacitated acrosome-intact human spermatozoa but showed binding to ∼63% of acrosome-reacted spermatozoa. The binding sites are observed in the region from the acrosome to the midpiece (Tsubamoto et al., 1999; Table 1). These observations are further confirmed by employing *E. coli* (38–645 aa)-/baculovirus (1–745 aa)-expressed recombinant human ZP2, wherein binding is observed only to the equatorial region of the acrosome-reacted human sperm (Chakravarty et al., 2008; Table 1). The native ZP2 purified from human eggs using immunoaffinity column binds over the equatorial region, post-acrosomal region and midpiece of the capacitated acrosome-intact human spermatozoa. In acrosome-reacted human sperm, ZP2 showed binding to the acrosome region as well as in the post-acrosome region, equatorial region and midpiece (Chiu et al., 2008b; Table 1). Incubation of capacitated human sperm with baculovirus-expressed recombinant human ZP2 does not lead to any significant increase in the AR as compared to spontaneous AR (Chakravarty et al., 2005; Figure 1). The role of human ZP2 as observed using either transgenic mice or purified native/recombinant proteins in human sperm-egg binding is summarized in Table 2.

### Human ZP3

#### Transgenic Mice Studies Revealed That ZP3 Has No Role in Sperm–Oocyte Binding

To understand the role of ZP3 in sperm-egg binding and fertilization, using gene-targeting and embryonic stem cell technologies ZP3 null mouse cell lines have been developed. The ZP3 null mice have follicles with germinal vesicle intact oocytes but completely lack a ZP matrix and have a disorganized corona radiata (Liu et al., 1996; Rankin et al., 1996). The females of these mice are sterile and the developmental potential of their oocytes is highly compromised. Subsequently, using transgenesis mouse lines have been developed wherein mouse ZP3 has been replaced with human ZP3 thereby expressing human-mouse chimeric ZP (human ZP3, mouse ZP1, and mouse ZP2). As compared to ZP3 null mouse, it led to the restoration of ZP matrix (Rankin et al., 2003). The eggs with mosaic ZP (mouse ZP1, mouse ZP2, and human ZP3) failed to bind human sperm. However, the mouse sperm bind to these eggs and these transgenic mice are fertile (Rankin et al., 1998). In another additional study, it has been shown that human sperm fail to bind to the transgenic zonae expressing human ZP3 in place of mouse ZP3 (Baibakov et al., 2012). These studies using transgenic mice suggested that ZP3 is not involved in human sperm-egg binding.

#### Native/Recombinant Human ZP3 Binds to Capacitated Human Spermatozoa and Induces AR

To study the role of human ZP3 in sperm–oocyte binding and induction of AR, it has been expressed using both prokaryotic as well as eukaryotic expression systems (van Duin et al., 1994; Dong et al., 2001; Bray et al., 2002; Chakravarty et al., 2005; Caballero-Campo et al., 2006; Jose et al., 2010; Chirinos et al., 2011). Recombinant human ZP3 expressed in human ovarian teratocarcinoma (PA-1) cells showed dose-dependent inhibition of *in vitro* sperm-ZP binding in a hemizona assay (Dong et al., 2001). Baculovirus-expressed recombinant human ZP3 (1-424 aa residues) labeled with FITC binds to either acrosomal cap or equatorial region of the capacitated acrosome-intact sperm whereas its binding is restricted to the equatorial region only in the acrosome-reacted human sperm (Chakravarty et al., 2008; Table 1). *E. coli*-expressed recombinant human ZP3 (23–348 aa residues) also showed similar binding profile with spermatozoa (Table 1). Native human ZP3 (∼58 kDa) purified from human eggs also binds to the acrosomal region, equatorial region and midpiece of the capacitated acrosome-intact spermatozoa. However, binding of native ZP3 was restricted to midpiece of the acrosome-reacted spermatozoa (Chiu et al., 2008b; Table 1).

By and large, *E. coli*-expressed recombinant human ZP3 (devoid of glycosylation) failed to induce AR when incubated with capacitated human sperm (Chakravarty et al., 2005, 2008). However, ZP3 expressed using either baculovirus expression system or mammalian expression system leads to a dose and time dependent increase in AR. Studies by various investigators have shown that approximately 10–28% capacitated human sperm undergo AR (AR in presence of ZP3 minus spontaneous AR) when treated with recombinant ZP3 (van Duin et al., 1994; Dong et al., 2001; Bray et al., 2002; Chakravarty et al., 2005, 2008; Figure 1). These observations have been further consolidated by showing that the native human ZP3 purified from human eggs also induces dose dependent increase in AR (Chiu et al., 2008a; Figure 1). In presence of native human ZP3 (25 pmol/ml), ∼37% human sperm undergo AR as compared to ∼10% spontaneous AR. The maximal AR was observed after 15 min of incubation with ZP3 (Chiu et al., 2008a). The ability of human ZP3 to bind to the capacitated human spermatozoa and induction of AR resides in its C-terminal fragment corresponding to 214–305 aa residues (Bansal et al., 2009; Figure 1). However, in another study, *E. coli*-expressed recombinant human ZP3 peptides corresponding to 22–176 and 177–348 aa residues do induce AR that could be inhibited by pertussis toxin, EGTA and pimozide- a T-type calcium channel blocker (Ni et al., 2007).

Initial classical studies done in mouse model showed ZP3 as the zona ligand for sperm-egg binding and induction of AR (Bleil and Wassarman, 1983; Beebe et al., 1992). Molecular genetic approaches based on exon swapping and site-directed mutagenesis revealed that the spermatozoa binding site of mouse ZP3 is encoded by exon 7 (Kinloch et al., 1995). Subsequently, *O*-glycans attached to Ser^332^ and Ser^334^ of ZP3 have been shown to be critical for its biological activity (Florman and Wassarman, 1985; Chen et al., 1998). However, mice deficient in glycosyl transferase were fertile suggesting that terminal galactose in alpha linkage is not critical for sperm-egg binding (Thall et al., 1995). Further studies showed that terminal galactose and *N*-acetylglucosamine of ZP3 are also not required for fertilization in mouse (Williams et al., 2007). *O*-linked glycosylation of Ser^332^ and Ser^334^ has not been observed in ZP3 purified from mouse eggs by mass spectrometry (Boja et al., 2003). Transgenic mouse lines with mutated serine residues of ZP3 have been developed (Liu et al., 1995). The eggs obtained from these transgenic mice showed normal sperm binding and mice were fertile when mated with normal male (Liu et al., 1995). Moreover, when ZP3 transgene with Ser^332^ to Ala^332^ and Ser^334^ to Ala^334^ mutations were introduced into ZP3 null mice, the resulting mice were fertile suggesting that sperm binding and AR took place in absence of normal mouse ZP3 (Gahlay et al., 2010).

### Human ZP4

#### Transgenic Animal Models Suggests No Role for Human ZP4 in Taxon-Specific Sperm-Egg Binding

To investigate the role of ZP4 in taxon-specific sperm-egg binding, transgenic mouse lines have been developed that expressed human ZP4 in addition to the mouse ZP1, ZP2, and ZP3 in the ZP matrix of growing oocytes (Yauger et al., 2011). Mating studies of transgenic mice expressing human ZP4 with normal male mice resulted in litters with a size comparable with non-transgenic female mice. *In vitro* sperm binding assay revealed that only mouse sperm and not human sperm bind to the mouse eggs expressing human ZP4 (Yauger et al., 2011; Baibakov et al., 2012). The binding of mouse sperm to the transgenic mouse eggs is comparable to the non-transgenic mouse eggs. These studies suggested that ZP4 may not be sufficient for taxon-specific sperm binding to the egg (Yauger et al., 2011). Recently, the relevance of ZP4 in maintenance of appropriate ZP matrix and *in vivo* preimplantation development of the blastocyst has been shown in *ZP4* knock out female rabbits. However, fertilization is not affected in the *ZP4* knock out animals (Lamas-Toranzo et al., 2019). Thus, observations from transgenic mouse as well as rabbit model suggest that ZP4 may not have role in sperm-egg binding.

#### Native/Recombinant Human ZP4 Binds to Human Spermatozoa and Induce AR

The initial evidence that ZP4 may have a role in human ‘sperm–oocyte’ binding came by using recombinant human ZP4 (Chakravarty et al., 2008; Table 1). Interestingly, binding of both ZP3 and ZP4 to the same capacitated spermatozoon has been demonstrated by using triple staining, suggesting that these two proteins may have different binding sites on the spermatozoon. Higher percentage of sperm showed binding of ZP4 to the acrosome region of the capacitated acrosome-intact sperm as compared to ZP3 (Chakravarty et al., 2008). In another independent study using immobilized baculovirus-expressed recombinant human ZP2, ZP3, and ZP4, highest number of sperm bound to ZP4 followed by ZP3 and ZP2 (Chirinos et al., 2011). Binding of native human ZP4 purified from human eggs to the entire head of the capacitated acrosome-intact human spermatozoa has also been demonstrated, which disappears after acrosome reaction (Chiu et al., 2008b; Table 1). Studies from the other species also suggest that ZP4 may have a role in sperm-egg binding. In rabbits, recombinant ZP4 binds to the acrosome of rabbit sperm (Prasad et al., 1996). Similarly, in porcine (Yurewicz et al., 1998) and bovine (Kanai et al., 2007) heterocomplexes of ZP3 and ZP4 are responsible for sperm-egg binding. The salient findings on the role of human ZP4 in sperm-egg binding as observed by using either transgenic animal models or native/recombinant protein are summarized in Table 2.

As the case with baculovirus-expressed human ZP1 and ZP3, incubation of capacitated human sperm with baculovirus-expressed human ZP4 also led to a dose dependent increase in AR (Chakravarty et al., 2005, 2008; Caballero-Campo et al., 2006; Figure 1). Dose dependent studies with baculovirus-expressed recombinant human ZP4 revealed that as low as 1 μg/ml protein induced a significant increase in AR. The significant increase in AR is observed after 15 min treatment with the recombinant protein, which plateau at 60 min. Approximately 22% sperm undergo AR in presence of recombinant ZP4 (20 μg/ml) as compared to ∼9% spontaneous AR under similar experimental conditions (Chakravarty et al., 2005). Further, incubation of capacitated sperm with purified native human ZP4 also led to a dose-dependent increase in AR (Chiu et al., 2008a; Figure 1). Approximately 29% capacitated human sperm undergo AR when treated with native human ZP4 (25 pmol/ml) as compared to ∼10% spontaneous AR. Interestingly, simultaneous treatment of capacitated human sperm with native ZP3 and ZP4 lead to an increase in the percentage of sperm undergoing AR to about 38% (Chiu et al., 2008a). Two monoclonal antibodies (MA-1662, MA-1671) generated against human ZP4 significantly inhibited baculovirus-expressed recombinant ZP4-mediated AR. Using recombinant peptides expressed in *E. coli*, the minimal epitope of MA-1671 was mapped to 126–130 aa residues and MA-1662 was mapped to 256–260 aa residues. These observations suggest that both *N*- and *C*-terminal parts of human ZP4 may be relevant for induction of AR (Xu et al., 2012).

## Role of ZP Glycans in Human Oocyte-Sperm Binding and Induction of Acrosomal Exocytosis

### Oocyte-Sperm Binding

Both glycans as well as protein–protein interactions have been shown to play a role during human oocyte–sperm binding. The observations that *E. coli*-expressed recombinant human ZP1, ZP3, and ZP4 bind to the capacitated acrosome-intact sperm support the hypothesis that protein–protein interaction is sufficient for binding of the ZP proteins to the spermatozoa (Chakravarty et al., 2008; Ganguly et al., 2010b). Homozygous mutant mice expressing *N*-terminal mouse ZP2 sperm binding domain with *N*-glycan site mutated (mo*ZP2^*N*83*Q*^*) in a *ZP2*^*Null*^ background showed normal mouse sperm binding to the oocytes suggesting that gamete recognition is glycan-independent (Tokuhiro and Dean, 2018). In the context of human sperm-egg binding, confirmation of these findings is still awaited from this group using gene-edited mice expressing human ZP2 *N*-terminal fragment with appropriate mutations in the glycosylation site(s).

On the contrary, mannose (which is present on human ZP glycoproteins) can inhibit *in vitro* human fertilization (Mori et al., 1989). Several oligosaccharides along with complex glycoconjugates bearing selectin-like ligands have been shown to be involved in human sperm-egg binding (Oehninger et al., 1998). In a hemizona binding assay, decrease in human sperm binding to human hemizona has been reported when sperm were pre-incubated with GlcNAc, mannose, fucose and galactose (Miranda et al., 1997). Periodate oxidation of human ZP suggested that sialic acid is also involved in human sperm-egg binding (Ozgur et al., 1998). Further, the sialyl-Lewis^*x*^ present on *N*- and *O*-linked glycans of human ZP plays an important role in human sperm–oocyte binding as pre-incubation of human sperm with it or its conjugate with bovine serum albumin or antibodies against it leads to inhibition of human sperm–oocyte binding (Pang et al., 2011). In another independent study, it has been shown that human ZP matrix is coated with high density of complex type *N*-glycans terminated with the sialyl-Lewis^*x*^ sequence. In hemizona assay, sialyl-Lewis^*x*^tetrasaccharide as well as neoglycoproteins terminated by sialyl-Lewis^*x*^ showed significant inhibition of human sperm-ZP binding (Clark, 2013). By using chemoenzymatically synthesized highly complex triantennary *N*-glycans again confirmed that sialyl-Lewis^*x*^ moiety is critical for human sperm-egg binding (Chinoy et al., 2018). On the other hand, eggs obtained from transgenic mice expressing human ZP2, mouse ZP1, and mouse ZP3 that bind human sperm do not show expression of sialyl-Lewis^*x*^ expression suggesting that it may not be critical for sperm-egg binding (Avella et al., 2014).

### Acrosome Reaction

As discussed in Section “Role of Human ZP Glycoproteins in Binding to the Spermatozoa and Induction of Acrosome Reaction,” *E. coli*-expressed recombinant ZP1, ZP3, and ZP4 by and large failed to induce AR in the capacitated human spermatozoa in spite of their binding to spermatozoa (Chakravarty et al., 2005, 2008; Ganguly et al., 2010b). Baculovirus-expressed recombinant human ZP3 and ZP4 deficient in *N*-linked glycosylation showed significant reduction in their ability to induce AR, whereas removal of *O*-linked glycosylation (by alkali treatment) has no adverse effect on their ability to induce AR (Chakravarty et al., 2008). Further, removal of *N*-linked glycosylation by *N*-glycosidase-F from native ZP3 and ZP4 purified from human eggs also significantly inhibited their ability to induce AR in capacitated human sperm as compared to untreated ZP3 and ZP4. As in case of baculovirus expressed recombinant proteins, removal of *O*-linked glycosylation of purified native ZP3 and ZP4 does not significantly decrease AR (Chiu et al., 2008a). These observations suggest that *N*-linked glycans of human zona proteins are more relevant than *O*-linked glycans for their ability to induce AR.

## Concluding Comments

Using eggs from transgenic mouse lines expressing human ZP1, ZP2, ZP3, and ZP4, it has been demonstrated that ZP2 plays an important role in human sperm-egg binding whereas ZP1, ZP3, and ZP4 may not be relevant for sperm-egg binding. On the contrary, using purified native and/or recombinant proteins, it has been shown that human ZP1, ZP3, and ZP4 binds to the capacitated human spermatozoa and induce AR. There may be various probable reasons for failure of human sperm to bind to the mouse transgenic zonae expressing human ZP1, ZP3, and ZP4. One of the possibilities is that mouse ZP matrix (∼8 μm) is thinner than human ZP matrix (12 μm). It is possible that the stoichiometry of human ZP1, ZP3, and ZP4 incorporated in the transgenic mouse zonae may be different as compared to human zonae. Further, the epitopes/domains of the human zona proteins responsible for human sperm binding may not be accessible in the transgenic mouse zonae. Though based on mobility on SDS-PAGE, human proteins expressed in transgenic mice zonae are post-translationally modified to a similar extent as observed in native human zona proteins, but the possibility of differential glycosylation of human zona proteins in the transgenic mouse remains. If glycosylation plays an important role in human sperm-egg binding, it may be a critical factor and needs to be investigated. Further, contribution, if any from oligosacharides of ZP glycoproteins in imparting species specificity of ‘sperm–oocyte’ binding also needs investigations. It may be imperative to complement the non-binding of human sperm to mouse transgenic zonae expressing human ZP1, ZP3, and ZP4 with positive results showing human sperm binding by improving assay conditions for sperm-egg binding to support that transgenic mouse model physiologically mimics human gamete recognition. On the other hand, use of individual native/recombinant human zona proteins to study their binding characteristics with spermatozoa and/or induction of AR has a limitation that physiologically, spermatozoa binds to the ZP matrix that has all the four glycoproteins present in ZP matrix. Further, analyses of mutations in the gene encoding human zona proteins from infertile women suggest that ZP1, ZP2, and ZP3 have a role in fertility and assembly of ZP matrix.

## Author Contributions

SG studied and critically analyzed the information available and wrote this review.

## Conflict of Interest

The author declares that the research was conducted in the absence of any commercial or financial relationships that could be construed as a potential conflict of interest.
